# Factors Associated with Workplace Psychological Violence Among Operating Room Professionals

**DOI:** 10.1192/j.eurpsy.2026.11320

**Published:** 2026-07-31

**Authors:** I. Kacem, S. Gmiza, C. Sridi, A. Gaddour, M. Gabsi, F. Chelly, A. Fki, A. Aloui, M. Maoua, M. Kahloul

**Affiliations:** 1 Occupational Medicine Department, Farhat Hached University Hospital; 2 University of Sousse, Faculty of Medicine of Sousse; 3 Research Laboratory LR19SP0; 4 Occupational Medicine Department, Sahloul University Hospital, Sousse; 5 Occupational Medicine Department, Ibn El Jazzar University Hospital, Kairouan; 6 Anesthesia and Intensive Care Department, Sahloul University Hospital, Sousse, Tunisia

## Abstract

**Introduction:**

Violence in the hospital setting is a growing problem worldwide. Given its serious and relevant consequences, many major international organizations rank it among the top global public health priorities.

**Objectives:**

To identify factors associated with psychological workplace violence among operating room professionals in Sousse.

**Methods:**

This is a descriptive, cross-sectional study conducted among operating room professionals at Sahloul University Hospital in Sousse over a three-month period (January, February, and March 2025). Data were collected using a simple questionnaire. French version of the LIPT (Leymann Inventory of Psychological Terror) questionnaire (14 items) was used to assess psychological violence.

**Results:**

The study population consisted predominantly of female healthcare workers (65%), with a mean age of 42.4 ± 5.6 years. Over half of the participants (52%) reported having experienced psychological violence in the workplace during the past 12 months. When assessed according to LPT, 57% of respondents met the criteria for exposure to psychological violence.The most frequently cited contributing factors to psychological violence were: excessive workload (reported by 83% of participants), lack of managerial support (65%), and poor intra-team communication (49%). However, no statistically significant associations were found between these factors and exposure to psychological violence in this sample. Regarding perceived impact, 53% of healthcare workers indicated that psychological violence negatively affected the quality of their professional performance.

**Conclusions:**

Psychological violence is prevalent among operating room staff in Tunisian hospitals. Addressing it requires targeted research and a multidisciplinary approach to build safer, more respectful workplaces.

**Disclosure of Interest:**

None Declared

